# Hsa_circ_0000345 regulates the cellular development of ASMCs in response to oxygenized low‐density lipoprotein

**DOI:** 10.1111/jcmm.15801

**Published:** 2020-08-31

**Authors:** Huifang Liu, Xiaowen Ma, Xin Wang, Xirui Ma, Ziming Mao, Jing Zhu, Fengling Chen

**Affiliations:** ^1^ Department of Endocrinology and Metabolism Shanghai Ninth People's Hospital Shanghai Jiao Tong University School of Medicine Shanghai China

**Keywords:** aortic smooth muscle cells, atherosclerosis, HIF‐1α, hsa_circ_0000345

## Abstract

The interaction between circRNAs and atherosclerosis has been extensively studied. However, more novel circRNAs need to be explored to help establish a perfect regulatory network. In the present research, hsa_circ_0000345 was demonstrated to regulate cellular development of oxygenized low‐density lipoprotein (ox‐LDL)‐treated aortic smooth muscle cells (ASMCs), which was closely related to the occurrence and progress of atherosclerosis. Ox‐LDL exposure remarkably decreased hsa_circ_0000345 expression in ASMCs. Transfection‐induced hsa_circ_0000345 overexpression activated cell viability (detected by an MTT assay) and restrained cellular apoptosis (analysed by flow cytometry) in the atherosclerosis cellular model. While down‐regulation of hsa_circ_0000345 reduced cell viability and promoted cell apoptosis. In addition, the data of the cell cycle distribution analysis and trans‐well assay indicated that cell cycle progression was arrested at the G1 phase while cell invasion was enhanced in ASMCs following treatment of ox‐LDL in the context of hsa_circ_0000345 OE plasmids. In addition, up‐regulation of hsa_circ_0000345 supported HIF‐1α at both the mRNA and protein level, and down‐regulation of hsa_circ_0000345 reduced HIF‐1α expression. Overall, the above findings revealed that hsa_circ_0000345 was a dramatic regulator of ASMCs proliferation, apoptosis and invasion in response to ox‐LDL treatment. Hsa_circ_0000345 was identified as a protector of cell viability during ox‐LDL induced cell development.

## 1 INTRODUCTION

Atherosclerosis is the principal basis of many cardiovascular diseases, such as coronary heart diseases, cerebral infarction and peripheral vascular diseases.^1^ The danger of other diseases caused by atherosclerosis has become a major concern. It was indispensable to understand the occurrence and development of atherosclerosis. The typical lesions of atherosclerosis includes arterial thickening, sclerosis, stenosis and atherosclerotic plaque formation.^2^, ^3^ Aortic smooth muscle cells (ASMCs) were inextricably involved in these processes.^4^ Consequently, exploring the cell fate of ASMCs is an important part of monitoring the development of atherosclerosis.

As an innovative classification of non‐coding RNAs, circRNA biogenesis relies on a canonical splicing mechanism and is affected by a combination of cis‐acting elements and trans‐splicing factors, such as heterogeneous nuclear ribonucleoprotein (hnRNPs) and SR proteins. Moreover, the functions of circRNA depend on acting as ‘sponges’ and regulating protein functions and, therefore, circRNAs participate in various physiological and pathological processes.^5^ In the cardiovascular system, it has been demonstrated that circRNAs have vital regulatory roles in angiogenesis, smooth muscle cell functions and cardiac injury responses.^6^, ^7^ Circ_ANRIL prevented ribosome biogenesis and pre‐rRNA processing by binding to PES1 (which is an essential 60S‐preribosomal assembly factor) and simultaneously activated P53 activity, eventually leading to the inhibition of vascular smooth muscle cell (VSMCs) proliferation and enhancement of cellular apoptosis. Therefore, circ_ANRIL was considered an anti‐atherosclerosis factor.^8^ Circ_Lrp6 was confirmed to be highly expressed in blood vessels and associated with vascular lesions. In addition, recent studies have shown that circ_Lrp6 regulated the proliferation and migration of VSMCs by sponging miR‐145.^9^ More importantly, circRNAs also participated in modulating cell fates in VMSCs under hypoxic conditions. For instance, silencing of circ_000595 decreased hypoxia‐induced VSMCs apoptosis by augmenting miR‐19a, which has been shown to arrest the cell cycle by impeding cyclinD1 expression.^10^, ^11^ cZNF292, another hypoxia‐induced circRNA, was shown to stimulate cell proliferation.^12^ All of the above data suggest the possibility of circRNA of atherosclerosis regulation.

Systemically expressed hypoxia inducible factors (HIFs) critically influence adaptive physiological responses including vascular remodelling. Many target genes are transcriptionally activated by HIF‐1α/HIF‐1β heterodimers containing vascular endothelial growth factor receptors. Complete knockout of HIF‐1α led to vascular regression and embryonic lethality. Studies have indicated that rescue of HIF‐1α expression could restore hind limb ischaemia in rats with diabetes and atherosclerosis.^13^ A recent phase I trial shown that patients with limb ischaemia might benefit from a constitutively active HIF‐1α hybrid.^14^ Therefore, increasing HIF‐1α expression may be capable of promoting vascular remodelling and improving ischaemic symptoms in atherosclerosis. Last but not least, circRNAs and HIF‐1α also closely interact. In hepatocellular carcinoma, has‐circ‐0046600 enhanced the expression of HIF‐1α through competitive binding with miR‐640.^15^ In addition, knockdown of circ_PIP5K1A suppressed HIF‐1α by promoting the sponging of miR‐600.^16^ In summary, circRNAs exerted dramatic effects on hypoxia and atherosclerosis; however, the role of circRNAs/HIF‐1α in atherosclerosis needs to be further explored.

Low‐density lipoprotein (LDL) is an important risk factor for atherosclerosis, and oxidative modified LDL (oxLDL) promotes atherosclerosis.^17^, ^18^ CircRNA microarray revealed hsa_circ_0000345 (chr11:77409531‐77413540) was down‐regulated in oxLDL induced human umbilical vein endothelial cells (HUVECs).^19^ In the current research, we investigated the role of hsa_circ_0000345 in the cellular development of oxLDL induced ASMCs to clarify potential mechanisms of atherosclerosis.

## 2 MATERIAL AND METHODS

### 2.1 Cell culture

Human aortic smooth muscle cells (ASMCs) were obtained from KeyGEN Bio TECH (Nanjing, China) and cultured in 10% foetal bovine serum (FBS)‐containing F12K medium with penicillin and streptomycin. The cell culture incubator chamber was maintained at 5% CO_2_ and 37°C. The cell culture medium was replaced every 2 days, and all experiments used cells within 15 passages.

### 2.2 Cell transfection

Circ_0000345 OE plasmids and si‐circ_0000345‐1 and si‐circ_0000345‐2 were constructed by Genechem (Shanghai, China), and transfection was achieved by Lipofectamine™ 2000 (Invitrogen, Carlsbad, CA, USA). Generally, we seeded the cells into 6‐well plates one day before transfection, and we incubated the cell until they reached 70% confluency. Lipo‐2000 and plasmids or siRNA (1:1) were diluted with 200 µL Opti‐MEM (Gibco, Gaithersburg, MD, USA) medium, and we mixed the liquids 5 minutes later. Subsequently, 400 µL of the mixture was added to each well after 20 minutes. After transfection for 6‐8 hours, the medium was replaced with normal medium. All experiments were performed the day after transfection.

### 2.3 RNA extraction and quantitative real‐time PCR (qRT‐PCR)

Total RNA was extracted from cells using TRIzol (Invitrogen) following the manufacturer's instructions. First‐strand cDNA was synthesized from total RNA using a reverse transcription system (Takara, China). The Sangon Biotech (Shanghai, China) provided all primers and GAPDH served as the reference gene. The primers used for qRT‐PCR were as follows: hsa_circ_0000345, 5’‐GTGGCAATTATCCCCAAACTGT‐3’ (forward), 5’‐GGTGGAAGAAGAGTCAACAGC‐3’ (reverse); GAPDH, 5’‐CAAGGTCATCCATGACAACTTTG‐3’ (forward), 5’‐GTCCACCACCCTGTTGCTGTAG‐3’ (reverse).

### 2.4 MTT assays

MTT assays were used to measure cell viability. ASMCs were digested, resuspended and seeded in 96‐well plates. After different treatments for the indicated days, cells were incubated with 40 µL of MTT (5 mg/mL) in each well, and the supernatant was discarded before 150 µL DMSO was added. After shaking for 10 minutes in the dark, the crystals were completely dissolved. Finally, the absorbance was detected using a microplate reader at 490 nm.

### 2.5 Flow cytometry for cellular apoptosis

Cellular apoptosis was detected using the Annexin V‐FITC/PI apoptosis detection kit (Bestbio, Shanghai, China) and flow cytometry (Beckman Coulter, Brea, CA, USA). After transfection and the indicated treatments, EDTA‐free trypsin (Beyotime, Shanghai, China) was used to digest the cells. We washed and collected cells twice by centrifugation at 225 *g* for 5 minutes. We then discarded the supernatants and resuspended cells with binding buffer (500 µL). Subsequently, 5 µL of Annexin V and 5 µL of propidium iodide (PI) were added into the cell suspension and incubated for 10 minutes. Finally, the percentage of specific cell populations was measured by flow cytometry to analyse the cellular apoptosis rate.

### 2.6 Flow cytometry for the cell cycle

Flow cytometry analysis was applied to detect the distribution of cell cycle by measuring the DNA content through Cell Cycle Analysis Kit (Beyotime, Shanghai, China). Briefly, cells were digested, washed and collected after the indicated treatment. Then, cells were fixed with 1 mL cold 70% ethanol at −20°C for at least 2 hours before measuring to stabilize the cell status. The suspension was centrifuged at 225 *g* for 5 minutes, and the ethanol was removed. We washed the cells with PBS and added 1 mL of hypotonic propidium iodide (PI) solution in the presence of 1% RNase A. After 30 minutes of incubation in the dark at 37°C, the cell cycle distribution was determined by flow cytometry.

### 2.7 Trans‐well assays

Pre‐treated ASMCs were collected and resuspended with non‐serum F12K medium. We pre‐coated the upper sides of the chambers of 24‐well trans‐well plates with Matrigel. Then, 200 µL of cell suspension was seeded into the upper chamber, and 600 µL of F12K with 10% FBS medium was added to the lower chamber. After 48 hours of incubation, we used a cotton swab to wipe the non‐migrating cells that remained in the upper chamber. The cells that migrated to the lower chamber were fixed with 95% ethanol for 20 minutes and then stained with 0.1% crystal violet for 15 minutes. Finally, we counted the cells under a light microscope after washing the cells twice with PBS.

### 2.8 Protein extraction and Western blot

ASMCs were collected after each treatment, and then, cold PBS and lysis buffer (PMSF: RIPA = 1:100) were applied to wash and lyse the cells, respectively. After 30 minutes, cell lysates were collected and centrifuged at 22 500 *g* for 30 minutes, followed by transferring the supernatants. Then, total protein was mixed with SDS‐PAGE loading buffer and separated with a 10% SDS‐PAGE gel. Subsequently, we electric‐blotted the target protein band onto PVDF membranes (Millipore Corp, Bedford, MA, USA). The membranes were blocked for 2 hours, washed with TBST and then incubated with diluted antibody against HIF‐1α (1:1000) and GAPDH (1:1000) overnight in a refrigerator (4°C). Finally, HRP‐conjugated secondary antibody (ZSGB‐Bio, Beijing, China) was added to the membranes for 1 hour at room temperature, and an ECL‐chemiluminescent kit (ECL‐plus, Hanover, NH, USA) was used to expose the immunoreactive blots. The relative protein expression was quantified by ImageJ 1.49 (National Institutes of Health).

### 2.9 Statistical analysis

All experiments were performed in triplicate. Statistical analyses were performed using Student's *t* test, one‐way ANOVA followed by Bonferroni's multiple comparison tests, as appropriate. Statistical data were performed using GraphPad Prism 6. *P* < 0.05 was considered statistically significant.

## 3 RESULTS

### 3.1 Ox‐LDL decreases hsa_circ_0000345 level

**FIGURE 1:**
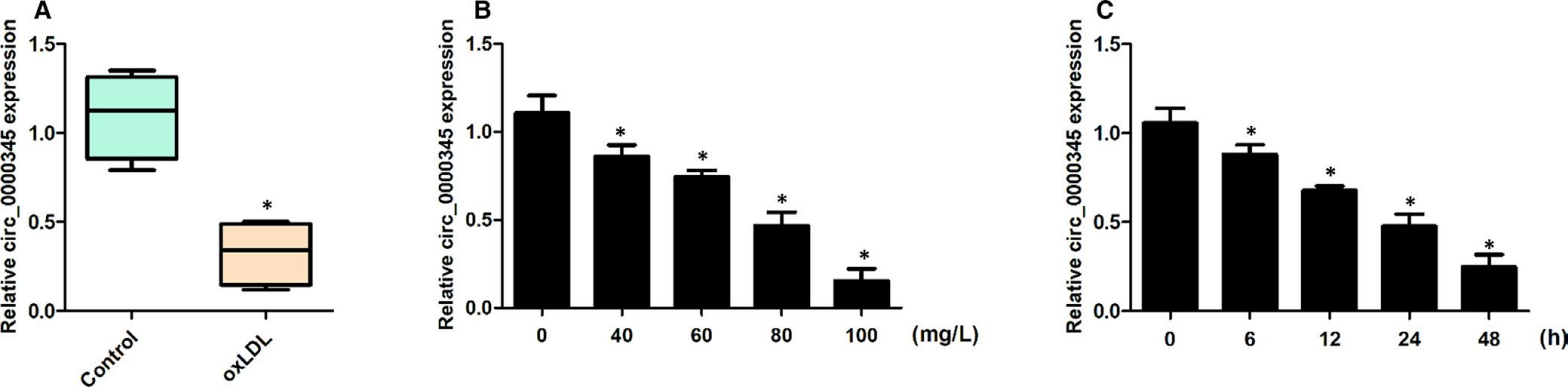
Hsa_circ_0000345 expression in response to ox‐LDL. (A) Relative expression of hsa_circ_0000345 in ox‐LDL‐treated ASMCs (oxLDL: treated with 100 mg/L oxLDL for 24 h) and normal ASMCs (Control: untreated). (B) ASMCs were treated with the indicated concentration (40‐100 mg/L) of ox‐LDL. (C) ASMCs were treated with ox‐LDL for the indicated time point (0, 6, 12, 24 or 48 h). The expression of hsa_circ_0000345 was analysed by qRT‐PCR. Values were presented as the mean ± SD of three independent experiments. *P < 0.05, significance versus the control group

In the present research, we conducted ox‐LDL to development an atherosclerosis cellular model.^19^, ^20^ To investigate whether hsa_circ_0000345 played a role in the response of ASMCs to atherosclerosis, we examined whether hsa_circ_0000345 level was altered in response to ox‐LDL. ASMCs were exposed to ox‐LDL at gradient concentrations (0, 40, 60, 80 or 100 mg/L). The RNA expression of hsa_circ_0000345 was decreased by ox‐LDL exposure (Figure 1A). Hsa_irc_0000345 had a concentration‐dependent reduction in expression (Figure 1B). Thereafter, 100 mg/L was used as an optimal concentration, and the level of hsa_circ_0000345 was measured at the indicated time points (0, 6, 12, 24 or 48 hours). As expected, a time‐dependent decline in hsa_circ_0000345 was observed in response to ox‐LDL (Figure 1C). The reduction of hsa_circ_0000345 started at 6 hours, which indicated that hsa_circ_0000345 was sensitive to ox‐LDL exposure in ASMCs and might play a key role in the early stage of atherosclerosis. In conclusion, hsa_circ_0000345 expression is inhibited by atherosclerosis. Therefore, it was necessary to explore the functions of hsa_circ_0000345.

### 3.2 Overexpression of hsa_circ_0000345 activates cell viability and restrains cell apoptosis

**FIGURE 2:**
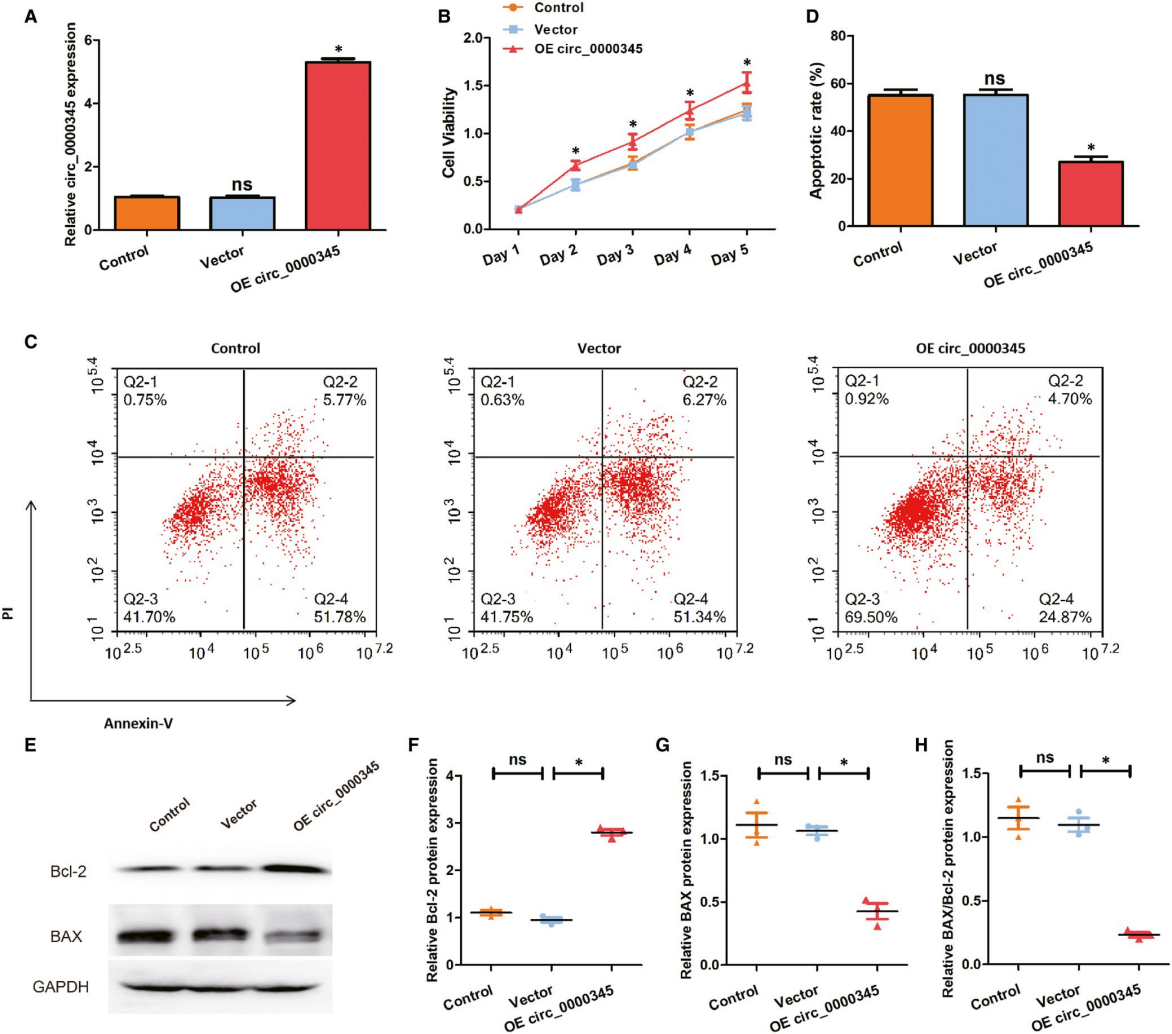
Hsa_circ_0000345 overexpression enhanced cell viability and inhibited cell apoptosis. (A) The relative RNA expression of hsa_circ_0000345 was determined by qRT‐PCR. (B) The cell viability was measured by MTT assay. (C and D) The cell apoptosis was detected by flow cytometry. (E‐H) Bcl‐2and BAX protein expression level were measured by Western blot analysis. Control represents cells without any transfection (exposed to 100 mg/L ox‐LDL for 24 h); vector represents cells transfected with empty vector (exposed to 100 mg/L ox‐LDL for 24 h); OE circ_0000345 represents cells transfected with hsa_circ_0000345 OE plasmids (exposed to 100 mg/L ox‐LDL for 24 h). Values were presented as the mean ± SD of three independent experiments. *P < 0.05, significance versus the control group. An empty vector was utilized as a negative control

To determine whether a specific role for hsa_circ_0000345 exists in modulating the destiny of ASMCs in response to ox‐LDL exposure, we constructed hsa_circ_0000345 OE plasmids and transfected them into ASMCs. As Figure 2A shows, the plasmids significantly enhanced the expression of hsa_circ_0000345. After transfection, MTT assays were used to detect cell viability of ASMCs. Enhancement of hsa_circ_0000345 improved cell viability as compared to controls (Figure 2B). In addition, we measured cellular apoptosis using flow cytometry after overexpression of circ_0000345. OE plasmid targeting hsa_circ_0000345 (apoptosis rate, about 30%) effectively reduced cellular apoptosis by approximately 30% in ox‐LDL pre‐treated ASMCs while a non‐targeting scrambled control plasmid (apoptosis rate, about 60%) had no appreciable effect on cellular apoptosis (Figure 2C and D). Figure 2E‐H indicated that overexpressing hsa_circ_0000345 significantly reduced relative BAX/Bcl‐2 protein expression in ox‐LDL pre‐treated ASMCs. These data demonstrate that hsa_circ_0000345 enhances cell viability and restrains cell apoptosis in ox‐LDL treated ASMCs.

### 3.3 Overexpression of hsa_circ_0000345 induces G1 phase cycle arrest and strengthens cell invasion

**FIGURE 3:**
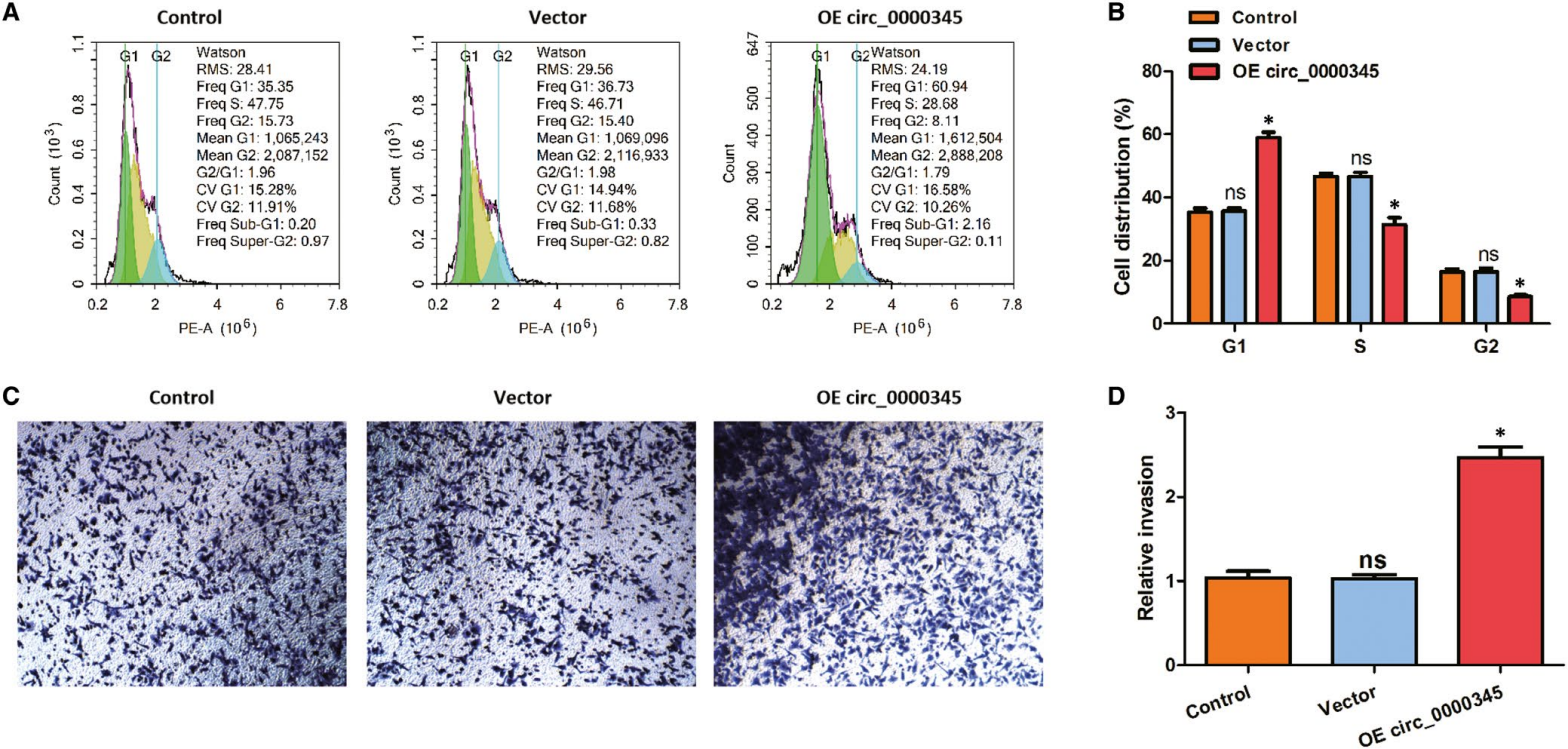
Hsa_circ_0000345 overexpression induced G1 phase cycle arrest and promoted cell invasion. (A and B) The distribution of each cell cycle was analysed by flow cytometry. (C and D) Cell migration was illustrated by trans‐well assays. Control represents cells without any transfection (exposed to 100 mg/L ox‐LDL for 24 h); vector represents cells transfected with empty vector (exposed to 100 mg/L ox‐LDL for 24 h); OE circ_0000345 represents cells transfected with hsa_circ_0000345 OE plasmids (exposed to 100 mg/L ox‐LDL for 24 h). Values were presented as the mean ± SD of three independent experiments. *P < 0.05, significance versus the control group

To further explore the effects of hsa_circ_0000345 in atherosclerosis, we assessed whether cellular proliferation or invasion was affected by hsa_circ_0000345 overexpression in ox‐LDL pre‐treated ASMCs. We utilized an analysis of cell cycle distribution to evaluate the cell proliferation. Compared with the control or vector, the proportion of cells in the G1 phase increased and the proportion of cells in the S or G2 phase decreased significantly after plasmid transfection (Figure 3A and B), indicating that hsa_circ_0000345 overexpression induced the cycle arrest at G1 phase. We also determined whether hsa_circ_0000345 overexpression via OE plasmids would have effects on cellular invasion. We conducted trans‐well assays in ASMCs and found that hsa_circ_0000345 overexpression significantly enhanced cell invasion (Figure 3C and D). These data suggest that hsa_circ_0000345 represses cellular proliferation and facilitates cellular invasion in ox‐LDL‐treated ASMCs.

### 3.4 Overexpression of hsa_circ_0000345 increased the protein expression levels of HIF‐1α

**FIGURE 4:**
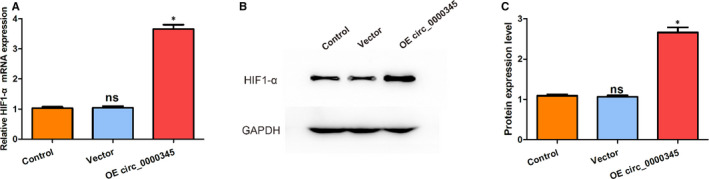
Expression of HIF‐1α mRNA and protein in response to hsa_circ_0000345 overexpression. (A) The relative mRNA level of HIF‐1α was measured by qRT‐PCR. (B and C) The relative protein level of HIF‐1α was detected through Western blot. Control represents cells without any transfection (exposed to 100 mg/L ox‐LDL for 24 h); vector represents cells transfected with empty vector (exposed to 100 mg/L ox‐LDL for 24 h); OE circ_0000345 represents cells transfected with hsa_circ_0000345 OE plasmids (exposed to 100 mg/L ox‐LDL for 24 h). Values were presented as the mean ± SD of three independent experiments. *P < 0.05, significance versus the control group. GAPDH was utilized as a negative control

To study the molecular basis that underlies the hsa_circ_0000345‐regulated the cellular development of ASMCs in response to ox‐LDL exposure, qRT‐PCR and Western blot were performed to measure the mRNA and protein level of HIF‐1α after transfection of hsa_circ_0000345 OE plasmids. The results illustrated that the mRNA level of HIF‐1α increased obviously following hsa_circ_0000345 overexpression (Figure 4A). Meanwhile, as shown by graphical and statistical analyses, an increase in protein expression was also observed (Figure 4B and C). As mentioned before, increasing HIF‐1α expression improves vascular remodelling and ischaemic symptoms in atherosclerosis. Hence, the above results suggest that hsa_circ_0000345 may ameliorate ischaemia and hypoxia during atherosclerosis and strengthen vascular remodelling.

### 3.5 Down‐regulation of hsa_circ_0000345 inhibits cell proliferation, strengthens cell apoptosis and decreased the protein expression levels of HIF‐1α

**FIGURE 5:**
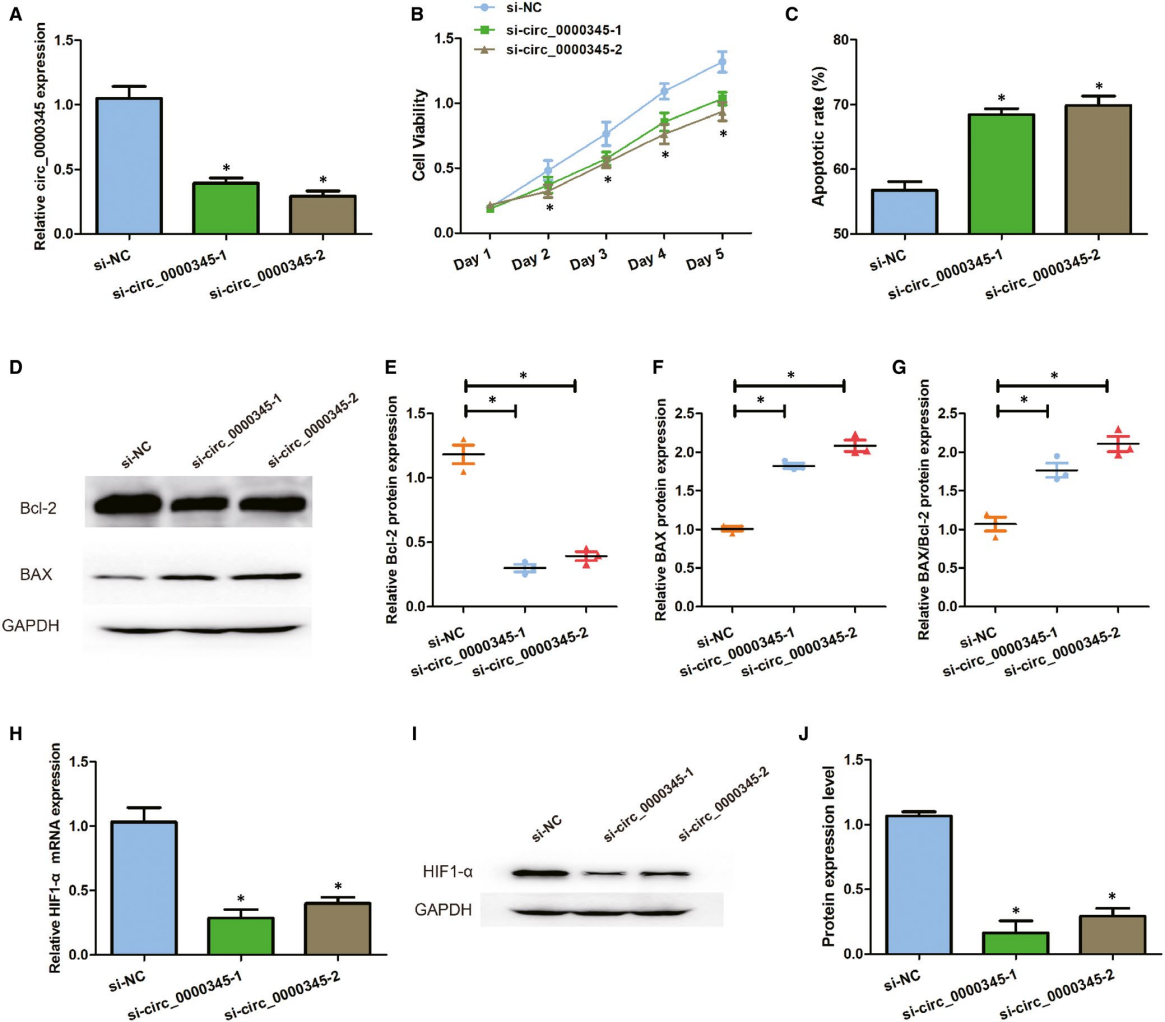
Hsa_circ_0000345 down‐expression reduced cell viability and expression of HIF‐1α and promoted cell apoptosis. (A) The relative RNA expression of hsa_circ_0000345 was determined by qRT‐PCR. (B) The cell viability was measured by MTT assay. (C) The cell apoptosis was detected by flow cytometry. (D‐G) Bcl‐2and BAX protein expression level were measured by Western blot analysis. (H) The relative mRNA level of HIF‐1α was measured by qRT‐PCR. (I and J) The relative protein level of HIF‐1α was detected through Western blot. si‐NC represents cells transfected with NC siRNA (exposed to 100 mg/L ox‐LDL for 24 h); si‐circ_0000345‐1 represents cells transfected with hsa_circ_0000345 siRNA‐1 (exposed to 100 mg/L ox‐LDL for 24 h); si‐circ_0000345‐2 represents cells transfected with hsa_circ_0000345 siRNA‐2 (exposed to 100 mg/L ox‐LDL for 24 h). Values were presented as the mean ± SD of three independent experiments. *P < 0.05, significance versus the control group. si‐NC was utilized as a negative control

We transfected si‐circ_0000345‐1 and si‐circ_0000345‐2 into ox‐LDL treated ASMCs. As Figure 5A shows, the siRNAs significantly reduced the expression of hsa_circ_0000345. MTT assays showed down‐regulation of hsa_circ_0000345 inhibited cell viability as compared to controls (Figure 5B). Flow cytometry results showed hsa_circ_0000345 silencing promoted cellular apoptosis from approximately 60% to 70% in ox‐LDL pre‐treated ASMCs (Figure 5C). Relative BAX/Bcl‐2 protein expression was also increased by down‐regulation of hsa_circ_0000345 (Figure 5D‐G). Down‐regulation of hsa_circ_0000345 also reduced the mRNA and protein expression of HIF‐1α (Figure 5H‐J). These results suggest that hsa_circ_0000345 silencing inhibited cell viability and HIF‐1α, promoted cellular apoptosis in ox‐LDL‐treated ASMCs.

## 4 DISCUSSION

Recently, the biological functions of circRNAs have been recognized due to the development of next‐generation sequencing and bioinformatics technology. As a non‐coding RNA family of remarkable significance, circRNAs have been shown to exert influence on several cardiovascular diseases with an atherosclerotic component.^21^, ^22^ In this report, we further confirm the important role of circRNAs in the development of atherosclerosis. Hsa_circ_0000345 was significantly decreased in a concentration‐ and time‐dependent manner by ox‐LDL induced ASMCs. Then, we describe the effects of hsa_circ_0000345 on cellular development of ASMCs in response to ox‐LDL exposure. As a consequence of hsa_circ_0000345 overexpression via OE plasmids, cell viability was increased gradually, and the early cellular apoptosis rate was significantly reduced. While the induction of hsa_circ_0000345 repressed cell viability and increased cell apoptosis. Furthermore, the enhancement of hsa_circ_0000345 arrested the cell cycle and impaired the transition of G1 and S; at the same time, cell invasion was dramatically increased. Taken together, hsa_circ_0000345 arrested the cell cycle at G1 stage and blocked apoptosis while promoting cell viability and cell invasion, demonstrating that hsa_circ_0000345 is a diversified regulator in ox‐LDL‐induced atherosclerosis.

Mounting researches have demonstrated that the development of atherosclerosis usually correlates with abnormal proliferation, migration and apoptosis of smooth muscle cells.^23^, ^24^ Regular angiogenesis and vascular physiological functions depend on well‐regulated cell proliferation and invasion of endothelial cells, VSMCs and monocyte/macrophages. During the progress of the primary lesions in atherosclerosis, monocytes migrate, proliferate and differentiate into macrophages in the arterial intima gradually, and macrophages swallow ox‐LDL, forming lipid‐laden macrophages (also called foam cells). Subsequently, foam cells and smooth muscle cells proliferate and eventually progress to advanced plaques.^25^, ^26^ Thus, exploring cell development mechanisms during ox‐LDL stress, for example, circRNA regulation, may help to uncover the distinctive and overlapping mechanisms of atherosclerosis, leading to effective targets. Consistent with the multiple effects of hsa_circ_0000345 in ASMCs, ox‐LDL is also a dual‐regulator in vascular cells, depending on the concentration used. Mild doses of ox‐LDL stimulate cell activation and inflammatory, while higher concentrations arrests cell growth and triggers cell apoptosis. In addition to regulating cell survival and apoptosis, ox‐LDL is also implicated in stimulating angiogenesis or anti‐angiogenesis responses, thereby contributing to each step of atherosclerosis progression. The biological influences of ox‐LDL, such as the balance between apoptosis and survival, depend on cellular systems that are regulated by non‐coding RNA. In our study, hsa_circ_0000345 was reduced after treatment with different concentrations of ox‐LDL. Therefore, it will be interesting to determine whether the multiple effects of hsa_circ_0000345 participate in the dual role of ox‐LDL.

The present work has shown that overexpression of hsa_circ_0000345 induced a reduction of HIF‐1α at both the transcriptional and translational level. The ability of HIFs to extensively modulate hypoxia has been appreciated for some time. Hypoxia is an inevitable manifestation of atherosclerosis due to the thickening of blood vessel walls.^27^ The most fundamental function of HIFs is angiogenesis management, which is an important risk factor in the progression of atherosclerosis. The mechanisms of angiogenesis have also been extensively studied. Migration inhibitory factor (MIF) was involved in foam cell transformation and vascular remodelling.^28^, ^29^, ^30^ In human umbilical artery smooth muscle cells (HUASMCs), HIF‐1α knockout triggered a down‐regulation of MIF, and inhibition of both MIF and HIF‐1α prevented the proliferation and migration of HUASMCs. In addition, hypoxia‐induced thrombospondin‐1 (THBS1) could be neutralized by HIF‐1 knockdown, and cell migration was simultaneously detained. The above data indicated the significant association between HIFs and the routine development of VSMCs. Meanwhile, many studies have documented a correlation between circRNAs and HIFs. In hepatocellular carcinoma, has_circ_0001730 inhibited tumorigenesis, development and metastasis by decreasing the expression of HIF‐1α.^31^ Has_circ_0010729 suppressed cell proliferation and promoted apoptosis, and has_circ_0010729 was coexpressed with HIF‐1α in HUVECs.^32^ Interestingly, our results confirmed that hsa_circ_0000345 enhanced HIF‐1α at the mRNA and protein level, showing an important connection between circRNAs and HIF. In addition, ox‐LDL induced a variety of transcription factors, including HIF‐1α, during the atherosclerosis development, which established the significance of ox‐LDL/circ_0000345/HIF‐1α signalling in the occurrence and development of atherosclerosis.

In summary, hsa_circ_0000345 could regulate the cellular development of ox‐LDL induced ASMCs. However, a detailed study of hsa_circ_0000345 will still be the focus of subsequent research.

## CONFLICT OF INTEREST

The authors declare that they have no conflict of interest.

## AUTHOR CONTRIBUTION

**Huifang Liu:** Data curation (equal). **Xiaowen Ma:** Data curation (equal). **Xin Wang:** Data curation (supporting). **Xirui Ma:** Data curation (supporting). **Ziming Mao:** Formal analysis (lead). **Jing Zhu:** Writing‐review & editing (equal). **Fengling Chen:** Conceptualization (equal); Writing‐review & editing (equal).

## Data Availability

The data used to support the findings of this study are available from the corresponding author upon request.
